# Negative Pressure Wound Therapy Literature Review of Efficacy, Cost Effectiveness, and Impact on Patients' Quality of Life in Chronic Wound Management and Its Implementation in the United Kingdom

**DOI:** 10.1155/2012/374398

**Published:** 2012-05-30

**Authors:** Diaa Othman

**Affiliations:** Burns, Plastics and Reconstructive Surgery, Nottingham University Hospitals NHS Trust, Nottingham NG5 1PB, UK

## Abstract

This is a paper reviewing the National Health Service (NHS) agenda in relation to the use of Negative Pressure Wound Therapy (NPWT) in chronic wound management and assesses the evidence behind it, its cost effectiveness and the outcome it has on patients' satisfaction and life style. Multiple studies over the last 10 years looking at clinical efficacy of NPWT with its cost effectiveness and the implementation of this service in the UK were reviewed. NPWT has showed a reasonable body of evidence to support its usage in chronic wounds with potential positive outcomes on finance and patients' satisfaction. However, the NHS system shows significant variations in the availability and implementation of this useful tool, depending on care providers and resources availabilities. The paper concluded that the NPWT can be a useful source of cutting down costs of chronic wound managements and saving money by its effect on expediting wound healing, which can address a part of the financial crises facing the NHS, however, has to be considered according to specific case needs. There should also be a national standard for the availability and indication of this tool to assure equal opportunities for different patients in different areas in the country.

## 1. Introduction

The National Health Service (NHS) represents itself as a unique system providing healthcare for all regardless of financial status. At the moment, it is reaching the point that patients' demands are overcoming NHS resources causing conflict in-between. Coombes [1] debated the best future for the NHS whether best to stay free at the point of use or be privatized. Moreover, the Secretary of State for Health proposed to abolish the NHS in England [2]. Bunt and Harris [3] claimed that the NHS needs to save *£*15 billion to *£*20 billion over the next few years and argued that significant savings can be achieved through radical patient-centred service redesign and more effective approaches to public behaviour change. The uses of financial rewards and pay-for-performance programs have been introduced to improve cost-effectiveness and quality of care, yet there remains scant evidence of the success of such initiatives from an economic perspective [4]. The focus on national targets in healthcare delivery has changed; the new focus is one of quality, innovation, productivity, and prevention (QIPP) programme [5, 6]. This has been described as “the new landscape in which we operate” [7]. 

Chronic wound management represents a considerable burden on health services and requires considerable manpower, frequent specialist consultation, and adjunct therapies; an important example of these adjunct therapies is the negative pressure wound therapy (NPWT), which was suggested to offer an important option for the advanced management of many wound types [8–10]. Manpower constitute a great portion of this cost. In their national UK audit, Drew et al. [11] suggested that nurses time accounts for 33–41% of the total cost of wound care. On the other hand, chronic wounds can also affect patients' ability to function in their environment, causing financial, social, and psychological consequences as well as affecting patients' Quality of Life (QoL) [12–14].

Patient's safety, effectiveness, and experience have been identified as quality domains in the NHS white paper [15]. It aims to put patients at the heart of the NHS by offering greater choice and control of services. The key is shared decision making, summed up by the phrase “no decision about me without me.” At the same time, it is recognised that the quality of health care cannot be allowed to decline; therefore, it must be subject to continuous improvement. The challenge faced by the NHS and practitioners within it is to improve the quality of care in an environment where the available resources are unlikely to keep pace with increasing demand [16]. Shorney [15] suggested that by using metrics, wound care services will be able to quantify the effectiveness of care provision and use this to argue for future resources and funding of such services.

This paper will consider the NHS agenda in relation to the use of NPWT in wound management, its cost effectiveness, its effects on patients' life, and healthcare demands and available resources.

## 2. Evidence behind NPWT Efficacy

NPWT benefits include rapid wound granulation, epithelialisation and contraction [17], reduction of dressing changes [18], reduced infection risk [19], reduced treatment costs [20], control of exudate [21], concurrent rehabilitation [22], and better patient tolerance [23].

The efficacy of NPWT was initially described by Morykwas et al. [24] and Morykwas [25]. Philbeck et al.'s [26] pioneering work studied 1,032 home healthcare patients with 1,170 wounds that failed to respond to previous interventions and were subsequently treated with NPWT and concluded NPWT to be efficacious and economical treatment modality. The conventional therapy cost was an estimation based on a study from 6 years before [27], which does not represent an accurate estimation as they did not allow for inflation.

Several studies have followed and identified faster healing times with NPWT when compared to moisturized saline gauze [28–32]. However, moisturized gauze is not an appropriate comparison where other modern dressings could have been compared to NWPT.

 Moues et al. [18] examined the total costs (hospitalization, nursing, and material) of 54 wounds. The mean was in favour of NPWT; NPWT had significantly higher material expenses (*P* < 0.0001), but significantly lower nursing expenses (*P* < 0.043). Schwein et al. [33] performed a retrospective analysis of 2288 pressure ulcers (PUs) in home health settings to examine both clinical and economic benefits of NPWT. A matched cohort of 60 NPWT patients showed lower rates of general hospitalisation (*P* < 0.05), wound problems (*P* < 0.01), and emergency admission (*P* = 0.01).

Llanos et al. [34] RCT on 60 patients with burns concluded improved skin graft take (*P* = 0.001) and shorter hospitalisation (*P* = 0.01) with NPWT. Blume et al. [35] conducted the largest multicentre RCT with 342 patients with diabetic foot ulcers (DFUs) comparing NPWT to alginate and hydrogel dressings and concluded that NPWT group had faster healing (*P* = 0.007), reduced secondary amputations (*P* = 0.035), and shorter hospitalisation period (89.5% versus 95.3%); however, it was not statistically significant. Trueman [36] pointed that the reduction of unnecessary hospital admissions opened the scope for the use smaller NPWT pumps allowing early patient discharge and management in the community. Potential benefits include freeing up hospital beds, reducing costs, improved patient satisfaction, and reduced hospital readmissions and nosocomial infections.

Meta-analysis is a principal method of cost-effectiveness analysis; however, the heterogeneity of such patients treated with NPWT makes it difficult to compare between different studies [37]. In their systematic review on NPWT, Vikatmaa et al. [38] studied 14 randomised clinical trials (RCTs) which included patients with PUs (two), posttraumatic wounds (three), DFUs (four), and miscellaneous chronic wounds (five). They reported that only two trials were classified as high quality studies. In all trials NPWT was at least as effective and in some cases more effective than the control treatment. They concluded NPWT to be a safe treatment, and serious adverse events have been rarely reported. Ubbink et al. [39] reviewed NPWT in 13 RCTs and concluded presence of a supportive evidence for the use of NPWT in the treatment of wounds.

These studies reflect an evidence that NPWT is efficient in treating wounds with improved clinical outcomes and should stimulate the healthcare system to provide such services and prevent it being obstructed by financial constrains. This motivated national and international committees to develop NPWT guidelines in wound care, such as the National Institute for Health and Clinical Excellence [40] report on NPWTs in open abdomen, which commented “The use of NPWT was initially confined to secondary care but this therapy is now provided in primary care, enabling earlier discharge for patients” meeting the targets of the DoH QIPP programme right care paper [41]. Williams [42] developed a practical document to support healthcare professionals and managers in developing a managed NPWT service to reduce costs and be able to access the service when needed and spare time and effort spent sourcing equipment before. Birke-Sorensen et al. [43] suggested the importance of developing an international consensus for NPWT recommendations and treatment variables.

## 3. NPWT Cost Analysis and Effect on Patients QoL

Three studies from 2006 [21, 44, 45] reported improved patients' QoL with NPWT applied to chronic wounds. Braakenburg et al.'s [21] blinded RCT (*n* = 65) compared NPWT to dressings (hydrocolloids, alginate, acetic acid, and sodium hypochlorite), although acetic acid and sodium hypochlorite are not recommended wound care products. NPWT had faster healing and greater wound size reduction and lesser time investment (*P* = 0.3 and *P* = 0.83, *P* = 0.04, resp.). Total costs were in favour of the dressings group, however, insignificant (*P* = 0.09). Although NPWT instruments and dressings are more expensive, their longer application on wounds and less frequent changing will reduce the total cost and the labour power and positively impacting on productivity. Vuerstaek et al.'s [44] prospective RCT (*n* = 60) reported quicker healing, faster wound preparation for grafting, and reduced costs with NPWT (*P* = 0.001, *P* = 0.005, *P* = 0.001, resp.); the major part of this cost difference was due to higher personnel costs and longer hospital stay in the dressings group caused by the slower healing. Both groups showed significant increase in patients' QoL and decrease in pain scores. Augustin and Zschocke [45] study (*n* = 176) measured outcomes before and after NPWT and reported significant (*P* < 0.001) increase in QoL and higher satisfaction. It is very important to include the patient in the decision making of the available treatment options as highlighted in the NHS agenda: “Shared decision-making will become the norm: no decision about me without me” [46]. Patients' involvement is an important point highlighted by the Right Care paper by the DoH [41] QIPP programme.

Searle and Milne's [47] literature review of the cost analyses of NPWT concluded that there is a strong evidence of NPWT for cost savings compared to conventional therapies. Abbotts [48] reported improved wound healing in all but one patient (*n* = 12). Concerns of most patients were the exudate smell from the canister, embarrassment, noise, and pain. On the other hand, these patients used to prepare the dressings before the arrival the nurse and they became confident with troubleshooting; they described patching air leaks and unblocking tubes. Changing dressings less frequently should reduce both exposure to contaminants and disruption to the wound healing process. Also, this level of patients' engagement can reduce time and effort spent by the staff and save the NHS further costs and free up nurses for other activities, leading to increased service productivity with a positive impact on the patient experience.

This represents an important aspect of the current DoH QIPP programme [49] to provide high level of care and yet save money by transforming community services. Dowsett et al.'s [16] cost analysis examined the savings made by implementation of the NPWT service in the community on 255 patients between 2009 and 2011. The cost per in-patient episode has been calculated comparing it to the secondary care; they concluded that by treating the patient in the community, there was cost saving of *£*4814 per patient. On the larger scale of wound care service delivered in the UK, the total savings could be very significant and address a large aspect of the current financial deficit in the NHS as well as help focusing the care of the patient closer to their home.

## 4. NPWT Demands and NHS Supplies

Achieving a balance between the NHS national agenda and patients most favourable outcomes is the key point. Mismatch between NHS demand and supply has always been a worry for care providers. The recent report by Lord Darzi [50] on transforming community services has followed his previous report [51] on providing high quality of care for all patients. He emphasised the importance of tissue viability and wound care in the community. He provided an example of “evidence-based practice” using NPWT and stressed on “getting the basics right every time”. This report remains a potential stimulus for the government to consider this issue as a high priority. White [52] claimed that the government had a low profile on tissue viability and wound care with developing risk on tissue viability nurses (TVNs) jobs due to financial pressure and urged the primary care commissioners to pay attention to the broad spectrum of wound care. The government started addressing this issue over the last 4 years, with the major focus on pressure ulcers problem, and already pledged to increase the influence of the nurses taking lead on this [53]. However, due to loss of collaboration between healthcare professionals, this may lead to clinical nurse specialist feeling “constrained” [54, 55].

The use of NPWT in home health settings remains relatively limited due to financial restrictions [16, 56, 57]. Some healthcare purchasing authorities in the UK provide a list of specific wounds where they consider NPWT is indicated [42], which sometimes limit individual decision making and can represent a collision between NHS demands and supplies [58]. This may create some challenges where issues such as accountability, shared resources, and assurance of care quality should be considered, including clinically effective, personally tailored, and safe level of service [59].

Harvard and Weston [60] reported that some healthcare professionals had difficulty in obtaining funding for some of the newer technologies, including NPWT. They examined the NHS tissue viability services in 173 trusts in England and concluded variable results, ranging from no dedicated provision (8.6%) to multidisciplinary tissue viability teams (31.9%). Only 33.6% of the trusts had outpatient NPWT service, with the other 66.4% having to admit patients for NPWT. Moreover, some healthcare professionals complained of a complicated approach to these services; 8% of the trusts reported problems obtaining NPWT funding. This financial and resources availability burden to manage patients with outpatients NPWT machines can cause significant inpatient costs, nosocomial infection risk, and financial and psychological concerns for patients themselves. 50% of the trusts claimed underfunding for their TVN and NPWT and compromising their outpatient NPWT facilities. This means that patients will receive different treatment levels depending on their post code and trust's catchment area, which may raise ethical inequity issues. Also, NPWT purchasing activity is predominated by NHS Trusts (devices) and NHS Supply Chain (consumables), a process that may compromise service delivery.

Searle and Milne [47] reported that the growing pressure on hospital beds has increased the use of NPWT in community settings. A recent publication from the DoH [61, 62] suggested that as a result of advances in tissue viability, more complex wound care can now be provided in the community setting and therapies such as NPWT should be commonplace and emphasised that tissue viability professionals should be appointed to direct service provision with high standards. A recent specialist opinion group review by Ousey and Milne [63] identified several issues related to the implementation and continuation of NPWT in primary care; this included untimely patients referrals requiring NPWT, lack of training of community patients and staff, complicated community funding pathways, and lack of co-ordination between secondary and primary care.

Adapting the NPWT use is a major step forward; NPWT is often perceived to be more expensive than advanced wound care; however, this perception may be based more on unit price considerations than on a comparison of the total treatment cost [63]. It is important to remember that there may be some cases where a new treatment is beneficial clinically and financially on the long term but is unaffordable to initialise the starting cost.

## 5. Conclusion

The NHS is trying to save money and yet provide the same quality of care. Wound management is a potential field where this could be addressed, through a wider use of NPWT.

There is a substantial body of clinical and economic evidence supporting NPWT in wound management, including early discharge and faster healing, fewer readmissions, better patients' QoL, and improved cost effectiveness meeting the DoH QIPP agenda. The type and quality of studies are mixed: alongside RCTs, there is evidence in *real world* clinical practice, in the form of retrospective clinical studies. However, further controlled RCTs are encouraged as well as recognition of personal clinical experiences. Beyond technical failures in applying the dressing, NPWT is safe and well tolerated by patients. However, it is essential to provide training and education and monitor its use in day-to-day practice. NPWT has evolved from large devices to smaller, more portable devices, which can allow a smoother transition from hospital to community. Currently, there is a substantial evidence to support lack of NPWT for both primary and secondary care due to financial constrains, which conflicts with the targets set by the DoH and QIPP reports. More focused attention by the NHS is warranted for such service to be rewarding clinically, financially, and socially. All patients should be assessed on an individual basis and have equality impact and risk assessments to ensure that every patient has the opportunity to benefit from NPWT, which is a valuable addition, if used appropriately to specific patients' case and health care provider experience.

These studies and others have provoked national and international committees to develop NPWT wound care guidelines. This should encourage the NHS to conduct studies to validate these results with implemented local evaluations and audits to support widespread adoption of NPWT in national wound care.

## References

[1] Coombes R (2008). The NHS debate. *British Medical Journal*.

[2] Pollock AM, Price D (2011). How the secretary of state for health proposes to abolish the NHS in England. *British Medical Journal*.

[3] Bunt L, Harris M The human factor; How transforming healthcare to involve the public can save money and save lives. http://www.nesta.org.uk/library/documents/the-human-factor.pdf.

[4] Walker S, Mason AR, Claxton K (2010). Value for money and the quality and outcomes framework in primary care in the UK NHS. *British Journal of General Practice*.

[5] Department of Health High Quality Care for All.DoH. http://www.dh.gov.uk/prod_consum_dh/groups/dh_digitalassets/@dh/@en/documents/digitalasset/dh_085828.pdf.

[6] Nicholson D Implementing the next stage review versions: the quality and productivity challenge. http://www.dh.gov.uk/prod_consum_dh/groups/dh_digitalassets/documents/digitalasset/dh_104255.pdf.

[7] Farrar M QIPP: quality, innovation, productivity and prevention.

[8] Banwell PE, Téot L (2003). Topical negative pressure (TNP): the evolution of a novel wound therapy. *Journal of Wound Care*.

[9] Morykwas MJ, Simpson J, Punger K, Argenta A, Kremers L, Argenta J (2006). Vacuum-assisted closure: state of basic research and physiologic foundation. *Plastic and Reconstructive Surgery*.

[10] Henderson V, Timmons J, Hurd T, Deroo K, Maloney S, Sabo S (2010). NPWT in everyday practice made easy. *Wounds International*.

[11] Drew P, Posnett J, Rusling L (2007). The cost of wound care for a local population in England. *International Wound Journal*.

[12] Franks PJ, McCullagh L, Moffatt CJ (2003). Assessing quality of life in patients with chronic leg ulceration using the medical outcomes short form-36 questionnaire. *Ostomy Wound Management*.

[13] Persoon A, Heinen MM, Van Der Vleuten CJM, De Rooij MJ, Van De Kerkhof PCM, Van Achterberg T (2004). Leg ulcers: a review of their impact on daily life. *Journal of Clinical Nursing*.

[14] Augustin M, Herberger K, Rustenbach SJ, Zschocke I, Blome C (2010). Quality of life evaluation in wounds: validation of the freiburg life quality assessment-wound module, a disease-specific instrument. *International Wound Journal*.

[15] Shorney R (2010). Equity and excellence: measuring the quality of wound care and tissue viability services. *Nursing Times*.

[16] Dowsett C, Davis L, Henderson V, Searle R The economic benefits of negative pressure wound therapy in community-based wound care in the NHS.

[17] Armstrong DG, Lavery LA (2005). Diabetic foot study consortium. Negative pressure wound therapy after partial diabetic foot amputation: a multicentre, randomised controlled trial. *The Lancet*.

[18] Mouës CM, van den Bemd GJ, Meerding WJ, Hovius SE (2005). An economic evaluation of the use of TNP on full-thickness wounds. *Journal of Wound Care*.

[19] Leininger BE, Rasmussen TE, Smith DL, Jenkins DH, Coppola C (2006). Experience with wound VAC and delayed primary closure of contaminated soft tissue injuries in Iraq. *Journal of Trauma*.

[20] Apelqvist J, Armstrong DG, Lavery LA, Boulton AJ (2008). Resource utilization and economic costs of care based on a randomized trial of vacuum-assisted closure therapy in the treatment of diabetic foot wounds. *American Journal of Surgery*.

[21] Braakenburg A, Obdeijn MC, Feitz R, Van Rooij IALM, Van Griethuysen AJ, Klinkenbijl JHG (2006). The clinical efficacy and cost effectiveness of the vacuum-assisted closure technique in the management of acute and chronic wounds: a randomized controlled trial. *Plastic and Reconstructive Surgery*.

[22] Park CA, Defranzo AJ, Marks MW, Molnar JA (2009). Outpatient reconstruction using integra and subatmospheric pressure. *Annals of Plastic Surgery*.

[23] Hurd T, Chadwick P, Cote J, Cockwill J, Mole TR, Smith JM (2010). Impact of gauze-based NPWT on the patient and nursing experience in the treatment of challenging wounds. *International Wound Journal*.

[24] Morykwas MJ, Argenta LC, Shelton-Brown EI, McGuirt W (1997). Vacuum-assisted closure: a new method for wound control and treatment: animal studies and basic foundation. *Annals of Plastic Surgery*.

[25] Morykwas MJ (1998). External application of sub-atmospheric pressure and healing: mechanisms of action. *Wound Healing Society Newsletter*.

[26] Philbeck TE, Whittington KT, Millsap MH, Briones RB, Wight DG, Schroeder WJ (1999). The clinical and cost effectiveness of externally applied negative pressure wound therapy in the treatment of wounds in home healthcare medicare patients. *Ostomy Wound Management*.

[27] Ferrell B, Osterweil D, Christenson P (1993). A randomized trial of low-air-loss beds for treatment of pressure ulcers. *Journal of the American Medical Association*.

[28] McCallon SK, Knight CA, Valiulus JP, Cunningham MW, McCulloch JM, Farinas LP (2000). Vacuum-assisted closure versus saline-moistened gauze in the healing of postoperative diabetic foot wounds. *Ostomy Wound Management*.

[29] Wanner MB, Schwarzl F, Strub B, Zaech GA, Pierer G (2003). Vacuum-assisted wound closure for cheaper and more comfortable healing of pressure sores: a prospective study. *Scandinavian Journal of Plastic and Reconstructive Surgery and Hand Surgery*.

[30] Mouës CM, Vos MC, Van Den Bemd GJCM, Stijnen T, Hovius SER (2004). Bacterial load in relation to vacuum-assisted closure wound therapy: a prospective randomized trial. *Wound Repair and Regeneration*.

[31] Etöz A, Özgenel Y, Özcan M (2004). The use of negative pressure wound therapy on diabetic foot ulcers: a preliminary controlled trial. *Wounds*.

[32] Moisidis E, Heath T, Boorer C, Ho K, Deva AK (2004). A prospective, blinded, randomized, controlled clinical trial of topical negative pressure use in skin grafting. *Plastic and Reconstructive Surgery*.

[33] Schwien T, Gilbert J, Lang C (2005). Pressure ulcer prevalence and the role of negative pressure wound therapy in home health quality outcomes. *Ostomy Wound Management*.

[34] Llanos S, Danilla S, Barraza C (2006). Effectiveness of negative pressure closure in the integration of split thickness skin grafts: a randomized, double-masked, controlled trial. *Annals of Surgery*.

[35] Blume PA (2008). Comparison of negative pressure wound therapy using vacuum-assisted closure with advanced moist wound therapy in the treatment of diabetic foot ulcers: a multicenter randomized controlled trial. *Diabetes Care*.

[36] Trueman P (2008). Cost-effectiveness considerations for home health V.A.C. therapy in the United States of America and its potential international application. *International Wound Journal*.

[37] Neubauer G, Ujlaky R (2003). The cost-effectiveness of topical negative pressure versus other wound-healing therapies. *Journal of Wound Care*.

[38] Vikatmaa P, Juutilainen V, Kuukasjärvi P, Malmivaara A (2008). Negative pressure wound therapy: a systematic review on effectiveness and safety. *European Journal of Vascular and Endovascular Surgery*.

[39] Ubbink DT, Westerbos SJ, Nelson EA, Vermeulen H (2008). A systematic review of topical negative pressure therapy for acute and chronic wounds. *British Journal of Surgery*.

[40] National Institute for Health and Clinical Excellence Clinical guideline IPG322; Negative pressure wound therapy for the open abdomen. http://guidance.nice.org.uk/IPG322/Guidance/pdf/English.

[41] Department of Health Right care case book. http://www.rightcare.nhs.uk/downloads/Right_Care_Casebook_Vol_1_Sept_2011_final.pdf.

[42] Williams K (2010). Template for management: developing a negative wound therapy service. *Wounds International*.

[43] Birke-Sorensen H, Malmsjo M, Rome P (2011). Evidence-based recommendations for negative pressure wound therapy: treatment variables (pressure levels, wound filler and contact layer) steps towards an international consensus. *Journal of Plastic, Reconstructive & Aesthetic Surgery*.

[44] Vuerstaek JD, Vainas T, Wuite J, Nelemans P, Neumann MHA, Veraart JCJM (2006). State-of-the-art treatment of chronic leg ulcers: a randomized controlled trial comparing vacuum-assisted closure (V.A.C.) with modern wound dressings. *Journal of Vascular Surgery*.

[45] Augustin M, Zschocke I (2006). Evaluation of patient benefits of ambulatory and stationary use of V.A.C. therapy. *Fortschritte der Medizin Originalien*.

[46] Department of Health Equity and excellence: Liberating the NHS. http://www.dh.gov.uk/prod_consum_dh/groups/dh_digitalassets/@dh/@en/@ps/documents/digitalasset/dh_117794.pdf.

[47] Searle R, Milne J (2010). Tools to compare the cost of NPWT with advanced wound care: an aid to clinical decision-making. *Wounds UK*.

[48] Abbotts J (2010). Patients’ views on topical negative pressure: ‘effective but smelly’. *British Journal of Nursing*.

[49] Department of Health Transforming Community Services Demonstrating and Measuring Achievement: Community Indicators for Quality Improvement. http://www.vast.org.uk/downloads/Health/TransformingCommunityServices.pdf.

[50] Darzi A Transforming community services: ambition, action, achievement. Transforming services for acute care closer to home. DoH. http://www.dh.gov.uk/en/Publicationsandstatistics/Publications/PublicationsPolicyAndGuidance/DH_101425.

[51] Darzi A High Quality Care for All. DoH. http://www.dh.gov.uk/prod_consum_dh/groups/dh_digitalassets/@dh/@en/documents/digitalasset/dh_085828.pdf.

[52] White RJ (2008). Tissue viability in tomorrow’s NHS. *Journal of Wound Care*.

[53] White R, Cutting K (2009). Second darzi report: mixed messages for tissue viability. *Wounds UK*.

[54] Austin L, Luker K, Ronald M (2006). Clinical nurse specialists as entrepreneurs: constrained or liberated. *Journal of Clinical Nursing*.

[55] Austin L, Luker K, Roland M (2006). Clinical nurse specialists and the practice of community nurses. *Journal of Advanced Nursing*.

[56] Millard D (2002). Larval therapy: a case for inclusion in the drug tariff. *Nursing Times*.

[57] Hampton S (2005). Vacuum therapy and its potential for wound healing in the community setting. *Journal of Community Nursing*.

[58] Cunningham JB, Kempling JS (2009). Implementing change in public sector organizations. *Management Decision*.

[59] Department of Health Best practice guidelines on joint working between the NHS and pharmaceutical industry and other relevant commercial organisations. DoH. http://www.dh.gov.uk/prod_consum_dh/groups/dh_digitalassets/@dh/@en/documents/digitalasset/dh_082569.pdf.

[60] Havard D, Weston C (2007). A snapshot of England’s tissue viability services. *Wounds UK*.

[61] Department of Health The Framework for Quality Accounts. DoH. http://www.dh.gov.uk/prod_consum_dh/groups/dh_digitalassets/documents/digitalasset/dh_105315.pdf.

[62] Department of Health Guidance on the routine collection of patient reported outcome measures (PROMS). http://www.dh.gov.uk/dr_consum_dh/groups/dh_digitalassets/@dh/@en/documents/digitalasset/dh_092625.pdf.

[63] Ousey K, Milne J (2010). Focus on negative pressure: exploring the barriers to adoption. *British Journal of Community Nursing*.

